# Effects of Preoperative Intravenous Clonidine in Patients Undergoing Cataract Surgery: A Double-Blind, Randomized Trial

**DOI:** 10.1155/2014/346549

**Published:** 2014-09-02

**Authors:** Ana Ellen Queiroz Santiago, Adriana Machado Issy, Rioko Kimiko Sakata

**Affiliations:** Federal University of São Paulo, Rua Três de Maio 61, Vila Clementino, 04044-020 São Paulo, SP, Brazil

## Abstract

*Objectives*. The aim of this study was to assess the effects of clonidine on intraoperative analgesia, sedation, intraocular and blood pressure, arrhythmia, and ischemia. *Methods*. Forty patients undergoing cataract surgery were allocated into two groups. They were monitored with Holter machine, the pupil was dilated, and 30 minutes later, 20 patients received clonidine (4 *µ*g/kg), while the other 20 patients were given a 0.9% saline intravenously. Twenty minutes later, 2% lidocaine gel was applied. There were assessed intraoperative analgesia, intraocular pressure, blood pressure, heart rate, and the occurrence of arrhythmias and myocardial ischemia. *Results*. Pain intensity was lower in G1 during the phacoemulsification, irrigation, aspiration, and intraocular lens implantation. The HR and BP were lower with clonidine. The IOP was lower with clonidine after 15 minutes and at the end of the surgery. Sedation was higher with clonidine. The incidence of arrhythmia was lower at the end of surgery with clonidine. The incidence of myocardial ischemia did not differ between the groups. *Conclusions*. Clonidine (4 *µ*g/kg) before a phacoemulsification reduced the intensity of pain during cataract surgery. It also induced sedation, reduction of BP, HR, and incidence of arrhythmia at the end of the surgery, and did not alter myocardial ischemia. This trial is registered with Clinicaltrials.gov NCT01677351.

## 1. Introduction

Cataract extraction requires analgesia, ocular akinesia, and is performed in the outpatient surgery [1].

Topical anesthesia is indicated in procedures involving the anterior part of the eye, lasting less than two hours. Sedation is performed so that the patients remain quiet and do not interfere with the procedure [2].

Sedation is adjusted to allow for the patient's cooperation during the procedure. Clonidine is one of the drugs used in this context because it causes sedation without inducing hypnosis and reduces the blood pressure (BP) and heart rate (HR) [3, 4]. The reduction of the intraocular pressure (IOP) represents an additional advantage of clonidine because it makes the eye surgery easier and reduces the frequency of complications, such as expulsion of the vitreous humor when the lens posterior capsule is broken during phacoemulsification [5].

Following the development of phacoemulsification, topical anesthesia became a well-accepted minimally invasive technique, although it does not provide an analgesia that is as efficacious as blocks [6]. The authors of a review concluded that clonidine promotes a reduction of postoperative pain [7]. This led us to conduct the study with the aim of assessing the effect of clonidine on the intraoperative analgesia, sedation, and intraocular pressure in patients subjected to cataract extraction under topical anesthesia. The expected main outcome was an improvement of the intraoperative analgesia. The secondary outcomes were better sedation and a reduction of the intraocular pressure (IOP) and arrhythmia.

## 2. Materials and Methods

The study was prospective, randomized, and double-blind and registered at ClinicalTrials.gov under code NCT01677351.

The patients were allocated into two groups of equal size. Random allocation to the groups was performed by means of numbers placed inside envelopes. Before the onset of anesthesia, a nurse or anesthetist who was not involved in the participant's later follow-up opened the envelope and prepared the syringes with clonidine or 0.9% saline solution as indicated. No other investigator who participated in the study or data collection was aware of the group to which each participant was allocated.

Following the approval by the Research Ethics Committee of Federal University of São Paulo (number 0609/01) and signature on the informed consent form, 40 patients who were 40 to 80 years of age, of both genders, with an ASA (American Society of Anesthesia) I, II, or III physical status, and subjected to cataract extraction by means of phacoemulsification under topical anesthesia at a tertiary university hospital were assessed. The following were excluded: patients with coronary heart disease, heart arrhythmias, or chronic pain; patients using analgesics 2 weeks before surgery; patients with tremors or cough; patients who had previous surgery; patients with claustrophobia, glaucoma, psychiatric disorders, or difficulty in communicating; and pregnant patients.

Monitoring included the following: electrocardiogram using a Holter device, arterial pressure (AP), heart rate (HR), oxygen saturation, and placement of a venous catheter followed by pupil dilation using 10% phenylephrine (two drops) and 1% tropicamide (two to eight drops). Thirty minutes later, the patients in clonidine group were given a clonidine solution (4 mcg*·*kg^−1^), while the patients in placebo group were given 0.9% saline solution. Twenty minutes later, topical anesthesia was performed with 2% lidocaine gel that was placed on the inferior conjunctival sac, and the patient was released for surgery.

Whenever it was needed, anesthesia was supplemented by instillation of 2% lidocaine (three to five drops) or an intracameral injection of 2% lidocaine (0.5 mL). These interventions were properly recorded.

Intraoperative analgesia was assessed on a numerical scale ranging from 0 to 10, before injection of the solution, after 5 and 15 minutes, blepharostat placement, incision of the cornea, capsulorhexis, phacoemulsification, irrigation and aspiration, and lens implantation, and at the end of surgery. Sedation was assessed by means of the Ramsay Sedation Scale ((1) patient is anxious; (2) patient is calm and awake; (3) patient is sleepy but opens eyes on command; (4) patient is sleeping and only responds to vigorous verbal stimuli; (5) patient is sleeping and responds to pain stimulation by glabellar tap; (6) patient does not respond to pain stimuli). The IOP was measured before and 15 minutes after administration of the solutions to both eyes and after the surgical procedure in only the eye not subjected to the surgery. The continuous electrocardiogram, Holter series 8500, was applied after the pupil dilation and was discontinued immediately at the end of surgery. The traces were analyzed by a cardiologist. Alterations of the ST segment and arrhythmias were analyzed.

The Instat Graph statistical software was used for the sample calculations and analysis of the results. A difference of 30% in pain intensity according to a numerical scale was considered clinically significant for 80% power and a 95% confidence interval (95% CI). Based on a preliminary evaluation, a standard deviation (SD) of 2.2 was estimated for the pain intensity score within the group of patients; the calculated sample size was 20 patients per group [8]. The statistical tests used were as follows: Student's *t*-test for age, body weight, height, IOP, HR, AP, and length of surgery and the Mann-Whitney test for HR, AP, pain intensity, sedation, IOP, and arrhythmia. Measures of central tendency, mean, and dispersion (standard deviation) were used. The level of statistical significance was established as <0.05.

## 3. Results

**Figure 1:**
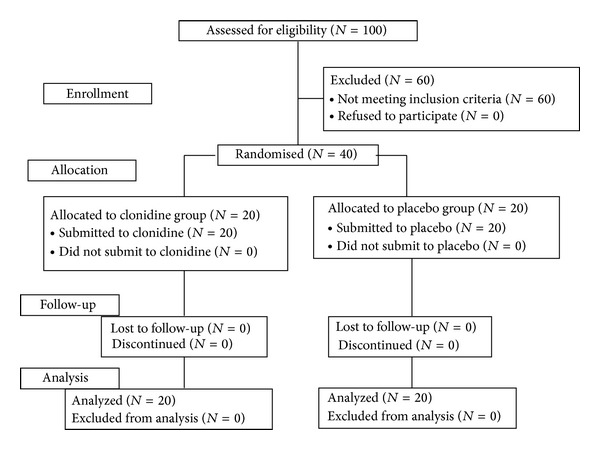
CONSORT flowchart.

The study sequence is described in a CONSORT diagram (Figure 1).

There was no difference between the groups regarding the demographic data (Table 1). The physical status was class ASA I for six patients in clonidine and eight patients in placebo group, and it was ASA II for eight patients in clonidine and 12 patients in placebo, without a significant difference between the groups (*P* = 0.7411; Mann-Whitney test). The surgery lasted 22 ± 9.9 min for clonidine and 24 ± 12 min for placebo (*P* = 0.05692; Student's *t*-test).

The intensity of pain exhibited a significant difference between the groups at the assessed time points (Table 2).

Sedation was greater in G1 at almost all the assessed time points (*P* < 0.05; Mann-Whitney test) (Table 3). Sedation increased in G1 between the onset (2.0 ± 0.0) and end (2.5 ± 0.6) of the surgery, which did not occur in G2 (2.0 ± 0.0 and 2.0 ± 0.0, respectively; Kruskal-Wallis analysis of variance (ANOVA test)).

The HR was lower when clonidine was used. The greatest HR measured was 69.4 ± 8.6 in G1 and 76.0 ± 18.5 in G2, while the lowest HR was 63.2 ± 11.1 in G1 and 69.3 ± 17.9 in G2.

At some time points, the systolic arterial pressure (SAP) was lower when clonidine was used (*P* < 0.05; Mann-Whitney test). At some time points, the diastolic arterial pressure (DAP) was lower when clonidine was used.

The IOP of the nonmydriatic eye exhibited a significant difference between the groups 15 minutes after injection of the solution (clonidine = 12.9 ± 3.1 and placebo = 15.5 ± 3.2; *P* = 0.0135; Mann-Whitney test) and at the end of surgery (clonidine = 12.5 ± 3.4 and placebo = 15.3 ± 2.7; *P* = 0.0065; Mann-Whitney test). The IOP of the mydriatic eye did not exhibit a difference between the groups.

The incidence of arrhythmia was higher in placebo only at the end of surgery, and there was no difference in the incidence of myocardial ischemia (Table 4).

## 4. Discussion 

Clonidine promoted a reduction in the intensity of intraoperative pain. It also promoted sedation and was associated with reductions in the BP, HR, and IOP. It did not reduce the incidence of arrhythmias or myocardial ischemia.

The intensity of pain decreased during the surgical steps that are considered to be uncomfortable. Although supplementary analgesia was not required in either group, the intensity of pain was lower when clonidine was used, thus showing that this drug is appropriate for the intended procedure.

In spite of its disadvantages, many patients prefer a topical anesthesia to a block due to the greater discomfort caused by the use of needles [6]. The analgesic effect is not as good as after blocks but there are fewer complications [6].

Complementary analgesia might be achieved with intracameral 1% lidocaine, which is effective as a supplement to topical anesthesia in cataract surgery via phacoemulsification [9]. In the present study, complementation was not required.

The safety of sedatives is related to the incidence of complications. The sedative and anxiolytic effects of clonidine, which are mediated through its binding to alpha-2 receptors [8], might avoid complications related to sympathetic stimulation [2]. In this study, no patients required supplementary sedation. In spite of the sedation induced by clonidine, the patients who received it were as awake at the end of surgery as the ones who were given a placebo, although the length of the procedure was shorter. For this reason, the time to hospital discharge did not differ.

Reduced IOP during cataract extraction facilitates the surgery performance and reduces the risk of complications. In the present study, the IOP of the patients given clonidine decreased at the end of the surgery (approximately 30 min), which was similar to another study [4].

Clonidine might reduce the HR and BP mediated by a decreased release of peripheral norepinephrine and a central sympatholytic effect [10]. In the present study, the HR was lower when clonidine was used; however, it remained within the normal range. There are no reports on significant alterations of the AP or HR when these mydriatics are used at their usual doses [8]. Bradycardia is a rare complication of clonidine even when used at high doses. The BP was lower when clonidine was used, but neither hyper- nor hypotension occurred with the dose used in the present study. Clonidine reduces the BP mainly when large doses are used. Bradycardia and hypotension were reported with higher doses (300 mcg) [7].

Clonidine might also reduce the incidence of myocardial ischemia in patients with coronary heart disease [11–13]. In the present study, the incidence of arrhythmia exhibited a difference only at the end of surgery, which suggests the antiarrhythmic action of clonidine. Arrhythmia before anesthesia might be related to the use of mydriatics and to anxiety. By inhibiting the sympathetic activity, clonidine might act as an anxiolytic and antiarrhythmogenic agent [14, 15].

The results of the present study suggest that clonidine at a dose of 4 *μ*/kg via an intravenous route promotes an analgesic effect and sedation appropriate for cataract extraction by means of phacoemulsification under topical anesthesia without altering the heart rate or the systemic arterial pressure. The results of the recent study further suggest that the use of clonidine reduces the incidence of arrhythmia.

## Limitations

Although no cardiovascular adverse effects were observed, the sample size of this study is too small to make any clinically relevant conclusions. In fact, the cataract population is usually old, has several comorbidities, and thus is at a very high risk of cardiovascular complications.

## Figures and Tables

**Table 1:** Demographic data.

	Clonidine (*n* = 20)	Placebo (*n* = 20)	*P*
Age (years)	64.3 (8.2)	65.5 (10.7)	0.69
Weight (kg)	65.8 (10.7)	70.0 (13.1)	0.28
Height (cm)	162.1 (8.5)	162.5 (8.8)	0.89
Gender (M : F)	7 : 13	9 : 11	0.75

Student's *t*-test.

**Table 2:** Intensity of pain, mean (SD).

Time points	Clonidine (*n* = 20)	Placebo (*n* = 20)	*P*
Control	0.4 (1.0)	0.2 (0.7)	0.77
5 min	0.3 (0.9)	0.2 (0.7)	0.79
15 min	0.3 (0.9)	0.2 (0.7)	0.79
Blepharostat	0.3 (0.9)	0.2 (0.7)	0.79
Incision of the cornea	0.3 (0.9)	0.2 (0.5)	0.99
Capsulorhexis	0.5 (1.1)	0.9 (1.3)	0.43
Phacoemulsification	1.4 (1.6)	3.9 (3.2)	0.02
Irrigation-aspiration	1.4 (2.0)	3.1 (2.7)	0.04
Lens implantation	1.7 (2.0)	3.6 (2.4)	0.01
End	1.1 (1.7)	1.8 (1.8)	0.20

Mann-Whitney test.

**Table 3:** Sedation (Ramsay Scale), mean (SD).

Time points	Clonidine (*n* = 20)	Placebo (*n* = 20)	*P*
Incision of the cornea	3.0 (0.5)	2.1 (0.3)	0.001
Irrigation-aspiration	2.8 (0.6)	1.9 (0.2)	0.001
Lens implantation	2.7 (0.6)	1.9 (0.2)	0.001

Mann-Whitney test.

**Table 4:** Incidence of arrhythmia and myocardial ischemia (percentage).

	Arrhythmia			Ischemia		
	Clonidine (*n* = 20)	Placebo (*n* = 20)	*P*	Clonidine (*n* = 20)	Placebo (*n* = 20)	*P*
Control	7 (46.6%)	6 (37.5%)	0.7224	2 (13.3%)	0 (0%)	0.23
5 min	3 (20%)	3 (18.7)	1.0000	0 (0%)	0 (0%)	NSA
15 min	2 (13.3%)	5 (31.3)	0.40	1 (6.6%)	0 (0%)	0.48
Blepharostat	4 (26.7%)	5 (31.3)	1.00	0 (0%)	0 (0%)	NSA
Incision of the cornea	0 (0%)	4 (25%)	0.10	0 (0%)	0 (0%)	NSA
Capsulorhexis	1 (6.6%)	4 (25%)	0.33	0 (0%)	0 (0%)	NSA
Phacoemulsification	4 (26.7%)	6 (37.5%)	0.70	0 (0%)	3 (18.7%)	0.23
Irrigation-aspiration	3 (20%)	5 (31.3)	0.69	0 (0%)	3 (18.7%)	0.23
Lens implantation	2 (13.3%)	6 (37.5%)	0.22	0 (0%)	3 (18.7%)	0.23
End	0 (0%)	5 (31.3)	0.04	0 (0%)	0 (0%)	NSA

NSA: not submitted to analysis; Fisher's exact test.
